# Systems Biology Approach Reveals Genome to Phenome Correlation in Type 2 Diabetes

**DOI:** 10.1371/journal.pone.0053522

**Published:** 2013-01-07

**Authors:** Priyanka Jain, Saurabh Vig, Malabika Datta, Dinesh Jindel, Ashok Kumar Mathur, Sandeep Kumar Mathur, Abhay Sharma

**Affiliations:** 1 Functional Genomics Unit, CSIR-Institute of Genomics and Integrative Biology, Council of Scientific and Industrial Research, Delhi, India; 2 Department of Surgery, Sawai Man Singh Medical College, Jaipur, India; 3 Department of Endocrinology, Sawai Man Singh Medical College, Jaipur, India; Medical University Hamburg, University Heart Center, Germany

## Abstract

Genome-wide association studies (GWASs) have discovered association of several loci with Type 2 diabetes (T2D), a common complex disease characterized by impaired insulin secretion by pancreatic β cells and insulin signaling in target tissues. However, effect of genetic risk variants on continuous glycemic measures in nondiabetic subjects mainly elucidates perturbation of insulin secretion. Also, the disease associated genes do not clearly converge on functional categories consistent with the known aspects of T2D pathophysiology. We used a systems biology approach to unravel genome to phenome correlation in T2D. We first examined enrichment of pathways in genes identified in T2D GWASs at genome-wide or lower levels of significance. Genes at lower significance threshold showed enrichment of insulin secretion related pathway. Notably, physical and genetic interaction network of these genes showed robust enrichment of insulin signaling and other T2D pathophysiology related pathways including insulin secretion. The network also overrepresented genes reported to interact with insulin secretion and insulin action targeting antidiabetic drugs. The drug interacting genes themselves showed overrepresentation of insulin signaling and other T2D relevant pathways. Next, we generated genome-wide expression profiles of multiple insulin responsive tissues from nondiabetic and diabetic patients. Remarkably, the differentially expressed genes showed significant overlap with the network genes, with the intersection showing enrichment of insulin signaling and other pathways consistent with T2D pathophysiology. Literature search led our genomic, interactomic, transcriptomic and toxicogenomic evidence to converge on TGF-beta signaling, a pathway known to play a crucial role in pancreatic islets development and function, and insulin signaling. Cumulatively, we find that GWAS genes relate directly to insulin secretion and indirectly, through collaborating with other genes, to insulin resistance. This seems to support the epidemiological evidence that environmentally triggered insulin resistance interacts with genetically programmed β cell dysfunction to precipitate diabetes.

## Introduction

Our understanding of genetic basis of disease risk has greatly improved in recent years owing to the advent of genome-wide association studies (GWASs) [1], [2]. The genes influencing common complex or multifactorial diseases and quantitative traits were largely unknown before GWASs came into being in the year 2006 [2], [3]. Results obtained from these studies suggest that multiple genetic architectures, including common genetic variants with small effects and rare variants with large effect sizes, underlie susceptibility to common diseases [1]. Nearly 1,300 GWASs covering more than 650 diseases and traits have been reported over the past several years [4]. A typical GWAS involves typing hundreds of thousands of single nucleotide polymorphisms (SNPs) in thousands of control and affected individuals, and identifying SNPs that differ significantly between the two groups in terms of allele frequency as disease or trait associated [5]–[7].

Type 2 diabetes mellitus (T2D) is a common complex disease whose pathogenic mechanisms are known to a considerable extent [8], [9]. Several organs including pancreatic islets, liver, skeletal muscle, adipose tissues, gut, hypothalamus and the immune system play a role in its pathogenesis [10]. Numerous multifactorial mechanisms that include genetic and environmental factors related to obesity are involved in the development of insulin resistance and impaired insulin secretion [8], [9]. Insulin resistance is associated with inactivity, obesity and ageing [8]. The insulin secreting pancreatic islet β cells respond to insulin resistance by enhancing their mass and metabolic function. T2D however develops when increase in insulin secretion by β cells is not able to keep pace with the increase in insulin resistance [8], [11]. The latter thus characterizes both prediabetic condition and T2D. Prediabetic insulin resistance state however does not always lead to diabetes; enhanced secretion of insulin by β cells compensates for deficient insulin action in a considerable proportion of prediabetic individuals who do not develop T2D. Though the inability of β cells to secrete enough insulin primarily typifies T2D, the dysfunction can also be demonstrated in normoglycemic subjects [12]. Therefore, derangements in both insulin secretion and insulin signaling, involved in the regulation of several processes including glucose uptake into cells, seem necessary but not sufficient in causing T2D. Based on epidemiological findings, it has been proposed that interaction between environmentally triggered insulin resistance and genetically programmed pancreatic β cell dysfunction leads to the development of T2D [12]–[14].

Over 20 major GWASs for T2D have been performed and several are underway at present [2]. A majority of identified loci have been found consistently across studies [15]. However, effect of the risk variants on continuous glycemic measures in nondiabetic subjects shows that T2D susceptibility is primarily mediated through perturbation of insulin secretion rather than insulin signaling [2], [12], [16]–[21]. Also, genes associated with T2D poorly represent established pathways of insulin signaling [12]. Global approaches to find statistically significant overrepresentation of functional categories in T2D associated genes have, although not provided clear evidence of the potential disease mechanisms, nonetheless identified enrichment of cell cycle regulation [2], [17], [19]. Considering that T2D associated genes representing cell cycle regulation are expressed in pancreatic islets, and that their disease association is mediated mainly through β cell dysfunction, the genetic evidence in the disease may seem to converge, to some extent, on insulin secretion [19]. Other than this limited convergence, the associated genes do not clearly confirm other known aspects of T2D pathophysiology including insulin signaling. This has led to the suggestion that either the disease is markedly heterogeneous or the critical aspects of disease pathophysiology are insufficiently captured by the presently available databases [2], [17], [19].

**Figure 1:**
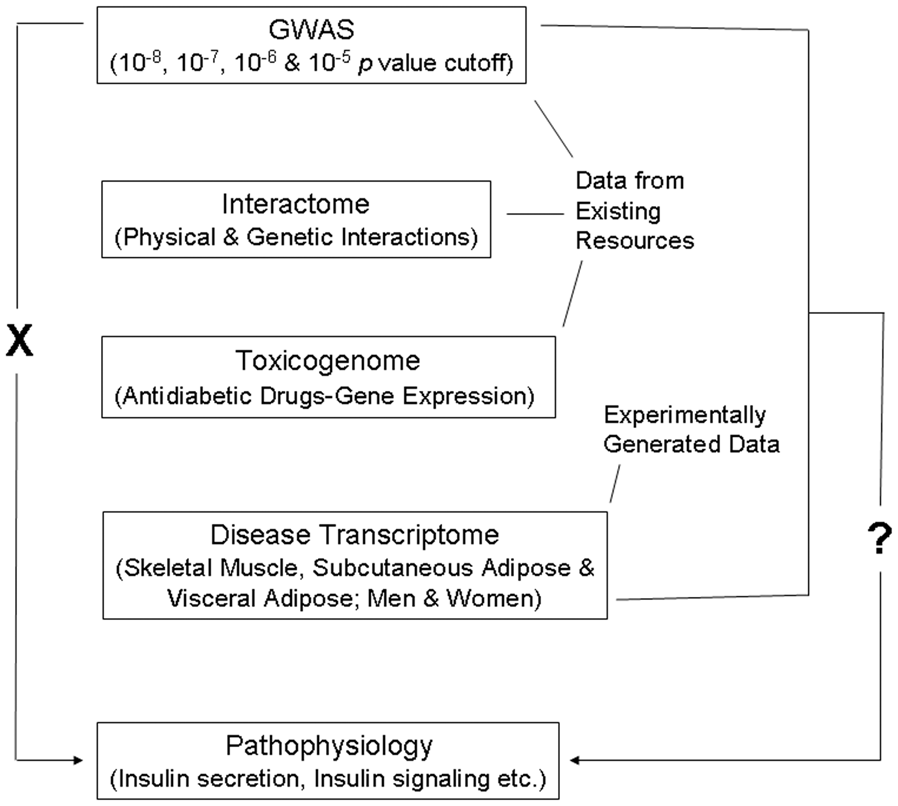
Schematic representation of the workflow. T2D GWAS genes do not directly relate (indicated by ‘X’ on the left side) to pathways associated with disease pathophysiology. Conspicuously, effect of identified risk variants on continuous glycemic measures in nondiabetic subjects chiefly explains only perturbation of insulin secretion, not insulin resistance. Further, the genes found as associated with the disease do not clearly relate to processes and pathways consistent with the known aspects of T2D pathophysiology. The main aim of the present study was to ask the question (indicated by ‘?’ on the right side) if GWAS data when considered in conjunction with interactome, toxicogenome and disease transcriptome data reveal genome to phenome correlation in T2D. Data available in public domain for GWAS, interactome and toxicogenome was used in the analysis. For disease transcriptome, new experimental data was generated. We specifically examined if interaction network of genes reported in T2D GWAS, genes showing altered expression after treatment with various antidiabetic drugs, and genes that are differentially expressed in insulin responsive tissues in male and female T2D patients do converge on insulin secretion, insulin resistance and other T2D associated pathophysiological pathways.

Genes identified in GWASs when evaluated in the context of complementary systems level data such as that related to protein-protein interactions and to and gene expression can provide insights into the mechanisms underlying pathogenesis of complex traits [22]–[24]. Here, we have combined these approaches toward deciphering genome to phenome correlation in T2D (**Figure 1**). Given that T2D GWAS genes do not directly relate to disease pathophysiology, our main aim was to examine if this genome to phenome correlation gap can be abridged by considering GWAS genes in conjunction with physical and genetic interaction, and gene expression data.

## Results

### GWAS Genes

**Figure 2:**
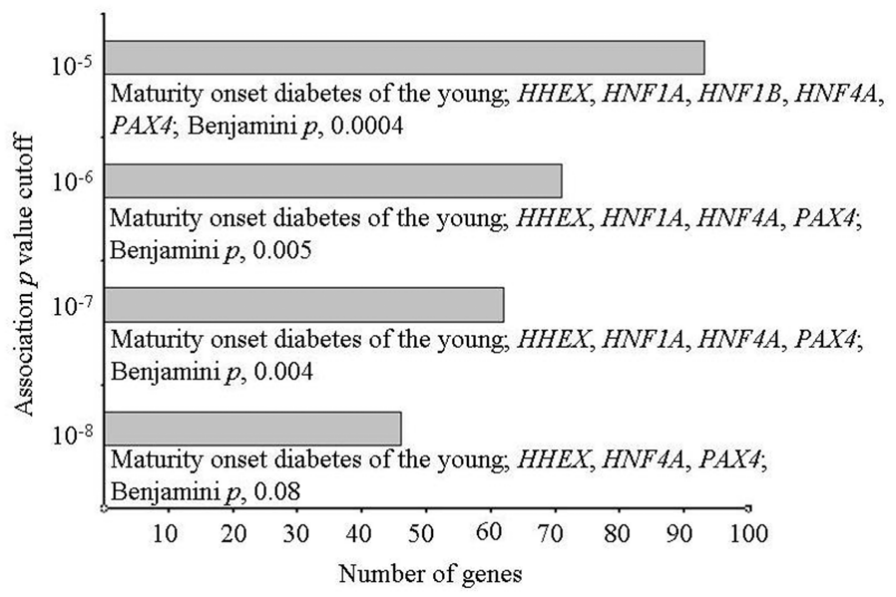
Enrichment of pathways in genes reported in T2D GWASs at various association *p* value thresholds. Genes at different *p* value cutoffs were examined for pathway enrichment. The pathway along with corresponding genes and enrichment *p* values are indicated. Note highly significant enrichment of Maturity onset diabetes of the young in genes reported at 10^−5^ *p* value threshold, dubbed “T2D genome” henceforth.

A catalog of SNP associations up to a *p* value cutoff of 1×10^−5^, a threshold commonly used for preliminary selection of SNPs in GWASs, exists in public domain [25]. As genes at this cutoff are considered meaningful for enrichment analysis [26], we retrieved genes reported in T2D GWASs at *p* value thresholds up to 10^−5^ (**Dataset S1**) and examined enrichment of Kyoto Encyclopedia of Genes and Genomes (KEGG) pathways therein. Maturity onset diabetes of the young (MODY), a Mendelian form of diabetes in T2D, was the only pathway that showed enrichment in this analysis (**Figure 2**). Whereas 10^−8^ cutoff genes showed enrichment of MODY only at a borderline significance, those at 10^−5^ threshold showed a robust overrepresentation. Of the five 10^−5^ cutoff genes in MODY, *HNF1A* and *HNF1B* were absent in 10^−8^ set while the latter alone was missing from 10^−7^ and 10^−6^ gene lists. These genes, like most of the other MODY genes, encode transcription factors that directly or indirectly affect expression of insulin and other proteins related to pancreatic β cell development and/or glucose metabolism [27]. Association of *HNF1A* and/or *HNF1B* with T2D has earlier been reported in candidate gene studies [28]–[30]. We thus focused on the gene list comprising 93 genes established from a GWAS cutoff of 10^−5^. Dubbed “T2D genome” henceforth, we used this gene set in subsequent analysis.

### Physical and Genetic Interaction Network

Next, we retrieved all the direct interactions, both physical and genetic, of T2D genome, called “T2D interactome” hereafter, from Biological General Repository for Interaction Datasets (BioGRID) (**Dataset S2**). Genes in T2D interactome was examined for pathway enrichment. Several of the enriched pathways relate to known aspects of T2D pathophysiology (**Table 1**). Other than insulin signaling and type II diabetes mellitus pathway that relates to both insulin secretion and insulin signaling, the other pathways such as ErbB, adipocytokine, Jak-STAT, chemokine, TGF-beta, Wnt, VEGF, Notch, MAPK, T cell receptor, B cell receptor, Toll-like receptor, p53 and mTOR signaling, and regulation of actin cytoskeleton have all been implicated in T2D [31]–[49]. Also, enrichment of cell cycle was consistent with previous global analysis of T2D associated genes [2], [17], [19]. Altogether, T2D interactome was found consistent with disease pathophysiology.

**Table 1:** Enriched pathways in T2D interactome.

Term	Corrected p value*
Pathways in cancer	4.52E-27
Prostate cancer	2.02E-17
Chronic myeloid leukemia	4.90E-17
B cell receptor signaling pathway	3.44E-15
Pancreatic cancer	1.38E-13
Glioma	3.24E-13
Focal adhesion	1.19E-12
Proteasome	3.38E-12
Acute myeloid leukemia	3.15E-12
Neurotrophin signaling pathway	1.39E-11
Insulin signaling pathway	2.57E-11
Small cell lung cancer	2.98E-11
T cell receptor signaling pathway	3.35E-10
ErbB signaling pathway	4.29E-10
Non-small cell lung cancer	4.09E-10
Colorectal cancer	7.45E-09
Cell cycle	9.14E-09
Fc epsilon RI signaling pathway	9.32E-09
Type II diabetes mellitus	2.00E-08
Adherens junction	4.16E-08
Melanoma	3.49E-07
Fc gamma R-mediated phagocytosis	3.46E-07
Adipocytokine signaling pathway	7.42E-07
Renal cell carcinoma	1.42E-06
Bladder cancer	1.72E-06
Jak-STAT signaling pathway	2.82E-06
Chemokine signaling pathway	3.28E-06
Endometrial cancer	3.65E-06
Notch signaling pathway	6.27E-06
TGF-beta signaling pathway	6.48E-06
Natural killer cell mediated cytotoxicity	6.30E-06
Aldosterone-regulated sodium reabsorption	7.51E-06
Thyroid cancer	1.01E-05
VEGF signaling pathway	1.44E-05
Progesterone-mediated oocyte maturation	8.87E-05
mTOR signaling pathway	9.80E-05
Leukocyte transendothelial migration	1.25E-04
Oocyte meiosis	1.57E-04
Pathogenic Escherichia coli infection	2.39E-04
Ubiquitin mediated proteolysis	3.04E-04
p53 signaling pathway	3.29E-04
Wnt signaling pathway	3.97E-04
NOD-like receptor signaling pathway	5.10E-04
MAPK signaling pathway	8.07E-04
Regulation of actin cytoskeleton	0.001
Toll-like receptor signaling pathway	0.002
Apoptosis	0.003
Epithelial cell signaling in Helicobacter pylori infection	0.004
Gap junction	0.01
RIG-I-like receptor signaling pathway	0.02
Tight junction	0.02
GnRH signaling pathway	0.02
Long-term depression	0.04

* Bejamini-Hochberg correction.

### Drug-gene Interactions

**Figure 3:**
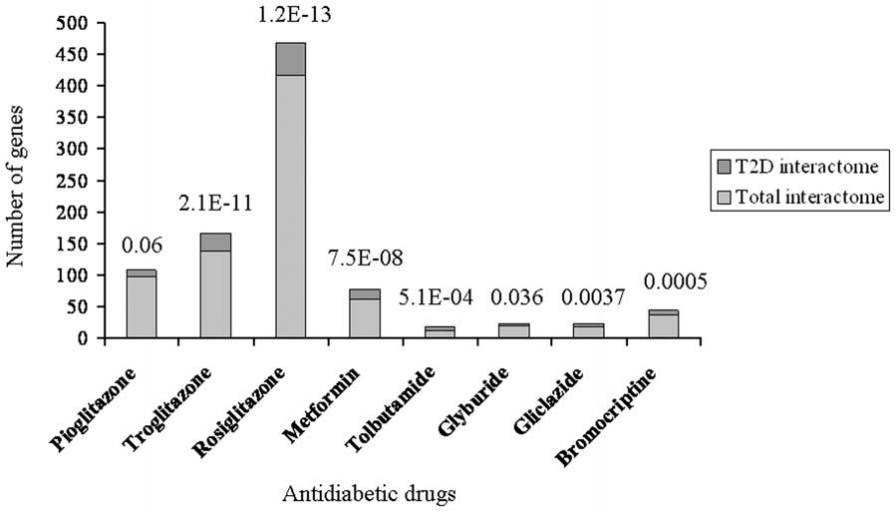
Overlap between antidiabetic drug interacting genes and T2D interactome. Compared to total interactome of 14,306 genes, the T2D interactome of 561 genes represent significantly greater number of antidiabetic drug interacting genes. A statistically significant overrepresentation was observed for all the drugs except pioglitazone in hypergeometric test with Bonferroni adjustment of *p* values for multiple hypotheses testing. Overrepresentation with a borderline significance was nonetheless observed even for pioglitazone. The adjusted enrichment *p* values are indicated.

We used a toxicogenomic approach to further investigate if T2D interactome represents pathophysiology related pathways including insulin signaling. The Comparative Toxicogenomics Database (CTD) documenting gene-environment relationships has been used previously towards understanding T2D etiology [24]. We searched for genes that interact with antidiabetic drugs using CTD (**Dataset S3**). We then examined if T2D interactome is enriched in genes interacting with these drugs. For enrichment analysis to be statistically meaningful, gene lists associated with only those drugs were considered which had a minimum of five genes in common with T2D interactome. Notably, significant enrichment or a trend for the same was observed for all the drugs tested (**Figure 3**). Further, genes interacting with all the antidiabetic drugs combined showed enrichment of various pathways relevant in T2D pathophysiology (**Table 2**), as observed previously for T2D interactome (**Table 1**). Cumulatively, the toxicogenomic analysis supported the importance of T2D interactome in unraveling genome to phenome correlation.

**Table 2:** Enriched pathways in antidiabetic drug interacting genes.

Term	Corrected p value*
Adipocytokine signaling pathway	4.16E-17
Pathways in cancer	3.30E-13
Prostate cancer	1.67E-10
Focal adhesion	4.23E-09
Bladder cancer	2.84E-08
Insulin signaling pathway	2.41E-08
p53 signaling pathway	2.51E-08
PPAR signaling pathway	3.05E-08
Pancreatic cancer	6.98E-08
Small cell lung cancer	6.79E-08
NOD-like receptor signaling pathway	2.84E-06
Drug metabolism	2.84E-06
Type II diabetes mellitus	4.93E-06
Colorectal cancer	5.63E-06
Retinol metabolism	6.51E-06
Metabolism of xenobiotics by cytochrome P450	6.30E-06
Glioma	1.32E-05
Cytokine-cytokine receptor interaction	1.44E-05
Chronic myeloid leukemia	4.34E-05
Melanoma	7.26E-05
mTOR signaling pathway	7.24E-05
Apoptosis	1.03E-04
Non-small cell lung cancer	1.09E-04
Linoleic acid metabolism	2.68E-04
Neurotrophin signaling pathway	2.75E-04
Toll-like receptor signaling pathway	2.67E-04
ErbB signaling pathway	2.94E-04
Thyroid cancer	3.24E-04
Cell cycle	7.72E-04
Acute myeloid leukemia	8.63E-04
Endometrial cancer	9.83E-04
MAPK signaling pathway	0.002
Allograft rejection	0.002
ABC transporters	0.003
Chemokine signaling pathway	0.004
ECM-receptor interaction	0.004
Arachidonic acid metabolism	0.006
Amyotrophic lateral sclerosis (ALS)	0.01
Jak-STAT signaling pathway	0.01
Steroid hormone biosynthesis	0.01
Fc epsilon RI signaling pathway	0.01
Renal cell carcinoma	0.01
Fatty acid metabolism	0.01
Pyruvate metabolism	0.01
Aldosterone-regulated sodium reabsorption	0.02
Intestinal immune network for IgA production	0.02
Type I diabetes mellitus	0.02
T cell receptor signaling pathway	0.02
Prion diseases	0.02
VEGF signaling pathway	0.02
Progesterone-mediated oocyte maturation	0.03
Fc gamma R-mediated phagocytosis	0.03
Glycerolipid metabolism	0.03
Citrate cycle (TCA cycle)	0.03
Gap junction	0.03
Glutathione metabolism	0.05
Starch and sucrose metabolism	0.05
TGF-beta signaling pathway	0.06

* Bejamini-Hochberg correction.

### Genome-wide Expression Profiling

**Figure 4:**
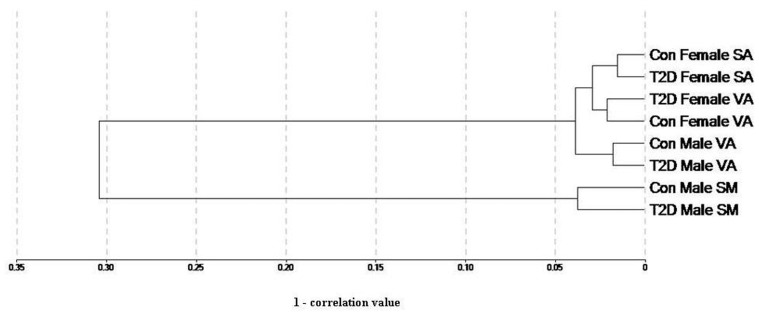
Dendrogram of samples based on gene expression profiling. Correlations between all the eight groups of samples analyzed in microarrays are plotted as a dendrogram. As expected, muscle and adipose form separate clusters. Also, in adipose cluster, subgroups of adipose type and gender are observed. Globally normalized data was used for constructing the dendrogram. Con, control subjects; T2D, diabetic patients; SA, subcutaneous adipose; VA, visceral adipose; SM, skeletal muscle.

**Figure 5:**
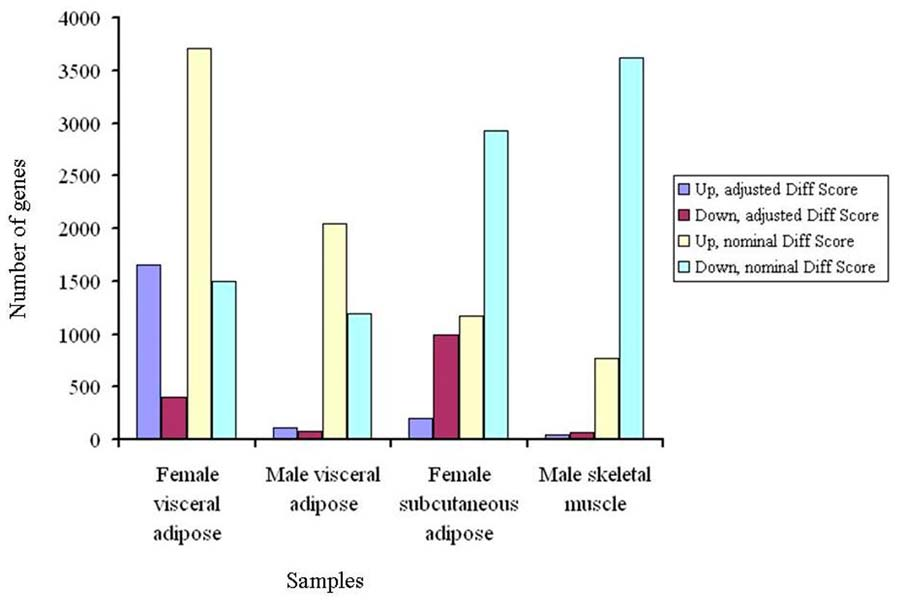
Numbers of up- and down- regulated genes in multiple insulin responsive tissues in T2D patients. Expression profiles of skeletal muscle, visceral adipose and subcutaneous adipose from male and/or female subjects were generated using Illumina HumanHT-12 v3 Expression BeadChip arrays that contain more than 25,000 annotated genes. IIlumina custom error model was used to identify up- and down- regulated genes in T2D as compared to controls, with or without Benjamini and Hochberg correction for multiple hypotheses testing. The differentially expressed genes were identified at ±13 Diff score threshold of Illumina custom algorithm, corresponding to a *p* value of 0.05.

**Figure 6:**
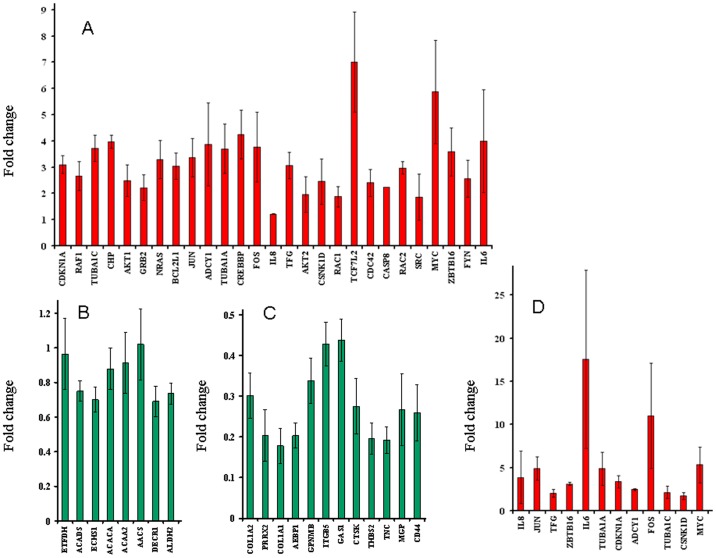
Validation of microarrays using qRT-PCR. Mean±S.E.M of fold change in gene expression in T2D patients, as compared to controls, is shown for (A) female visceral adipose, (B) male visceral adipose, (C) male skeletal muscle, and (D) female subcutaneous adipose. Fold change was calculated for two to six technical replicates, each representing three biological replicates. The source of RNA used in qRT-PCR analysis was same as in microarray profiling. A total of 47 genes were used for validation. The rationale behind the subsets of genes selected was two fold. One, the genes should represent, wherever applicable, one or more enriched pathways (**Table 3**) in a given condition. Second, the genes should maximally represent those which are differentially expressed at adjusted *p* value cutoff (**Dataset S4**) in more than one condition, so that validation of microarrays can be examined more widely. The selected genes, besides others, covered all the pathways that were enriched in the above microarray gene lists. Notably, up- or down- regulation observed in qRT-PCR, shown in red and green, respectively, was consistent with microarrays, for all comparisons.

Next, we generated microarray expression profiles of skeletal muscle, visceral adipose and subcutaneous adipose from male and/or female T2D patients (**Figure 4**). For each tissue tested, expression profiles were generated from three diabetic and three non-diabetic individuals. Also, each individual was profiled four times. The differentially expressed genes between diabetic and nondiabetic control groups were identified (**Figure 5** and **Dataset S4**). Significant genes at adjusted *p* value were subjected to pathway enrichment analysis. For all comparisons except female subcutaneous adipose, these genes showed enrichment of one or more pathways (**Table 3**). Female visceral adipose showed a large number of enriched pathways including ErbB, Wnt, MAPK, T cell receptor, B cell receptor and Toll-like receptor signaling, and regulation of actin cytoskeleton, which, as mentioned above, have all been implicated in T2D. Enrichment of valine, leucine and isoleucine degradation in male visceral adipose is also consistent with metabolomic studies in T2D [50], [51]. Similarly, enrichment of ECM-receptor interaction in male skeletal muscle is in consonance with known changes in the composition of the extracellular matrix in insulin-resistant muscle [52]–[57]. Quantitative real time PCR (qRT-PCR) confirmed differential expression in the same direction, or a trend for that, of all the genes tested to validate microarray results (**Figure 6**). The genes used in qRT-PCR validation represented all the enriched pathways (**Table 3**) besides others. These results demonstrated the robustness of our genome-wide expression profiling.

**Table 3:** Enriched pathways in differentially expressed genes between people with and without T2D.

Term	Corrected p value*
**Female visceral adipose**	
Neurotrophin signaling pathway	1.8E-04
Renal cell carcinoma	0.002
Thyroid cancer	0.002
Pathogenic Escherichia coli infection	0.002
Prostate cancer	0.002
Fc gamma R-mediated phagocytosis	0.002
Non-small cell lung cancer	0.002
Pathways in cancer	0.002
Chemokine signaling pathway	0.003
Acute myeloid leukemia	0.004
Endometrial cancer	0.01
Glioma	0.01
Focal adhesion	0.01
Adherens junction	0.01
Wnt signaling pathway	0.01
MAPK signaling pathway	0.01
B cell receptor signaling pathway	0.02
Chronic myeloid leukemia	0.02
ErbB signaling pathway	0.02
Bladder cancer	0.02
Regulation of actin cytoskeleton	0.03
Colorectal cancer	0.03
Toll-like receptor signaling pathway	0.04
T cell receptor signaling pathway	0.04
Gap junction	0.04
**Male visceral adipose**	
Valine, leucine and isoleucine degradation	3.0E-05
Propanoate metabolism	0.01
**Male skeletal muscle**	
ECM-receptor interaction	0.007

* Bejamini-Hochberg correction.

### Convergent Pathway

Although we found enrichment of several pathways consistent with T2D pathophysiology in our microarray analysis, insulin signaling was conspicuous by its absence in the list of overrepresented pathways (**Table 3**). Statistical corrections may be overly conservative to the point of being counterproductive in interpreting microarrays with genetic knowledgebases [58]. We thus explained this counterintuitive result by arguing that application of such corrections for identifying differentially expressed genes in microarrays and for pathway enrichment analysis of these genes may be together responsible for causing false negatives in our results. Since our main goal was to decipher genome to phenome correlation, we examined if T2D interactome (**Dataset S2**) is enriched for differentially expressed genes in various samples at unadjusted *p* value threshold (**Figure 5** and **Dataset S4**), called “T2D transcriptome” from now on. Enrichment will be expected if there is a genome to phenome correlation. Also, if enrichment is indeed observed at this level then genes overlapping between T2D interactome and T2D transcriptome will be expected to overrepresent pathways consistent with the disease pathophysiology.

**Figure 7:**
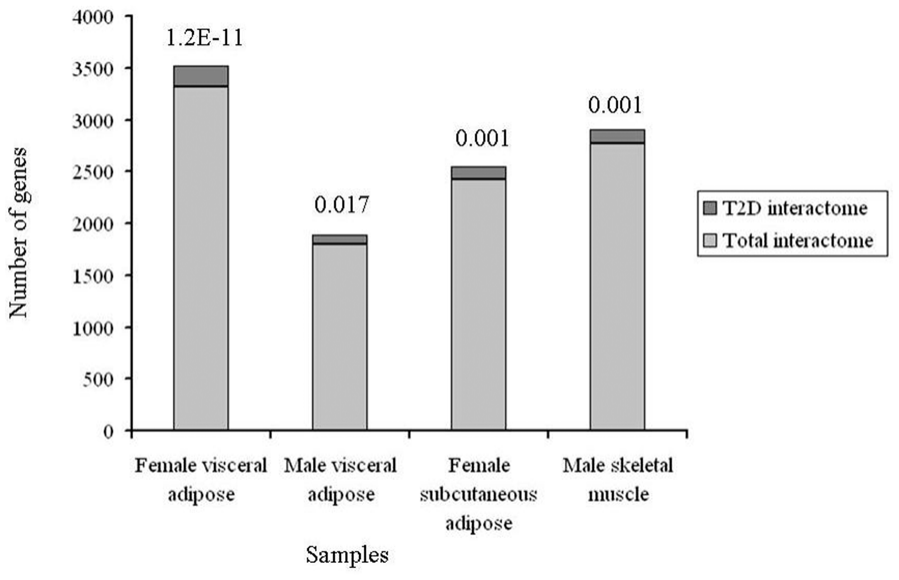
Overlap between T2D interactome and T2D transcriptome. Compared to total interactome of 14,306 genes, the T2D interactome of 561 genes represent significantly greater number of genes differentially expressed at unadjusted *p* value threshold in microarray profiles, dubbed “T2D transcriptome” from now on. Bonferroni adjusted hypergeometric distribution *p* values for the overlaps are indicated.

**Figure 8:**
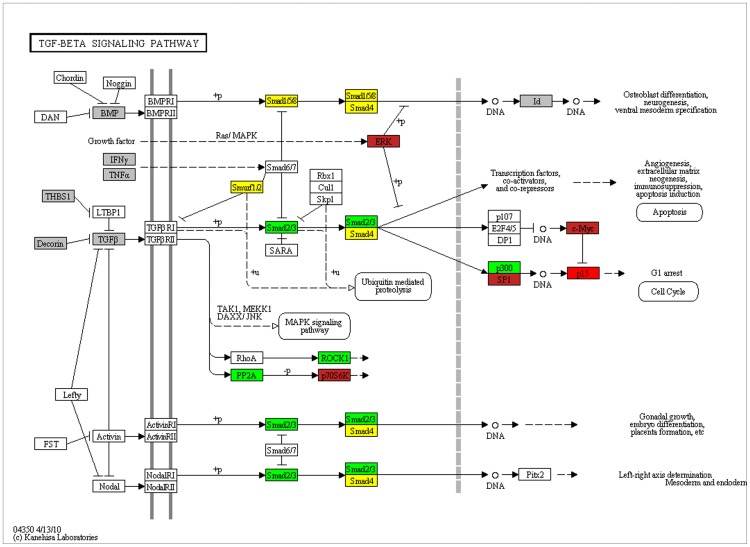
Genome to phenome pathway of TGF-beta signaling. T2D genome, T2D interactome, T2D transcriptome and antidiabetic drug interacting genes are mapped on to KEGG pathway for TGF-beta signaling. Red: genome, interactome and transcriptome; brown: interactome, transcriptome and antidiabetic drug interacting genes; green: interactome and transcriptome; yellow: interactome; grey: antidiabetic drug interacting genes.

Indeed, the overlaps between interactome and transcriptome gene sets were found to be statistically significant even after adjusting the *p* values for multiple testing (**Figure 7**). Also, the overlapping genes were enriched in T2D pathophysiology related pathways including insulin signaling in female visceral adipose and male skeletal muscle (**Table 4**). In male visceral adipose and female subcutaneous adipose, insulin signaling was however not enriched. Twelve pathways were common to all tissues and both genders (**Table 4**). The common pathways were related to cancer, cell cycle, adherens junction, focal adhesion, pathogenic *Escherichia coli* infection and TGF-beta signaling. To examine known role of these pathway(s) in T2D pathophysiology, the PubMed was searched using the key words “diabetes AND type AND 2 AND insulin AND (signaling OR action OR resistance OR sensitivity) AND (secretion OR pancreatic OR islets OR beta)” in combination with term(s) representing each of these common pathway. The retrieved abstracts and/or papers and the references therein were manually curated to identify a pathway that is known to play a role in both insulin secretion and insulin signaling related function. Literature search revealed that of these pathways TGF-beta signaling is particularly notable in that it is clearly known to play a crucial role in both insulin signaling as well as in insulin gene expression and pancreatic β cell function [36], [59]–[61]. Given this, we examined if removal of TGF-beta signaling genes from the list of T2D interactome-T2D transcriptome commonality genes negatively affect enrichment of insulin signaling in female visceral adipose and male skeletal muscle. There were 10 and 7 TGF-beta signaling genes in female visceral adipose and male skeletal muscle (**Table 5**), in that order. We removed these genes from 200 and 137 commonality genes (**Figure 7**) in T2D interactome-female visceral adipose and T2D interactome-male skeletal muscle comparisons and examined pathway enrichment in the resulting gene sets. Remarkably, compared to complete gene sets, 190 and 130 genes that remained after removing TGF-beta genes, in that order, showed less pronounced enrichment of pathways in general, and insulin signaling in particular (**Table 6**). Importantly, T2D genome includes one of the TGF-beta signaling genes, *CDKN2B*. This gene is associated with T2D in diverse populations at genome-wide significance level (**Dataset S1**). Cumulatively, our analysis identified TGF-beta signaling as a connecting link between genome and phenome in T2D (**Table 7** and **Figure 8** **)**.

**Table 4:** Enriched pathways in T2D interactome-T2D transcriptome intersection genes.

Term	Unadjusted p value	Corrected p value*
**Female visceral adipose**		
Pathways in cancer	3.91E-20	4.07E-18
Prostate cancer	8.27E-17	5.77E-15
Chronic myeloid leukemia	8.08E-15	2.81E-13
Acute myeloid leukemia	4.79E-12	1.24E-10
Glioma	1.77E-11	3.68E-10
Pancreatic cancer	1.38E-10	2.40E-09
Non-small cell lung cancer	3.69E-10	5.48E-09
Proteasome	8.70E-10	1.13E-08
Small cell lung cancer	1.38E-09	1.59E-08
Adherens junction	3.96E-09	4.12E-08
Melanoma	1.35E-08	1.28E-07
Thyroid cancer	1.87E-08	1.62E-07
Endometrial cancer	3.87E-08	3.10E-07
Jak-STAT signaling pathway	2.09E-07	1.55E-06
B cell receptor signaling pathway	2.40E-07	1.67E-06
Neurotrophin signaling pathway	3.19E-07	2.08E-06
Bladder cancer	6.33E-07	3.87E-06
Colorectal cancer	8.58E-07	4.96E-06
Cell cycle	2.09E-06	1.15E-05
T cell receptor signaling pathway	2.18E-06	1.14E-05
Insulin signaling pathway	5.27E-06	2.61E-05
Focal adhesion	8.16E-06	3.86E-05
ErbB signaling pathway	8.54E-06	3.86E-05
Renal cell carcinoma	5.11E-05	2.22E-04
Chemokine signaling pathway	5.42E-05	2.25E-04
Pathogenic Escherichia coli infection	7.18E-05	2.87E-04
Fc gamma R-mediated phagocytosis	1.11E-04	4.27E-04
Fc epsilon RI signaling pathway	1.21E-04	4.50E-04
Notch signaling pathway	1.41E-04	5.04E-04
TGF-beta signaling pathway	2.82E-04	9.77E-04
Wnt signaling pathway	3.43E-04	0.001
Oocyte meiosis	3.74E-04	0.001
Tight junction	4.63E-04	0.001
Progesterone-mediated oocyte maturation	0.001	0.004
Adipocytokine signaling pathway	0.001	0.004
p53 signaling pathway	0.001	0.004
Long-term depression	0.001	0.004
mTOR signaling pathway	0.002	0.005
GnRH signaling pathway	0.003	0.008
Melanogenesis	0.003	0.008
Dorso-ventral axis formation	0.003	0.008
Toll-like receptor signaling pathway	0.003	0.009
NOD-like receptor signaling pathway	0.004	0.01
Natural killer cell mediated cytotoxicity	0.006	0.01
Gap junction	0.006	0.01
Ubiquitin mediated proteolysis	0.007	0.01
MAPK signaling pathway	0.01	0.03
Regulation of actin cytoskeleton	0.02	0.04
Aldosterone-regulated sodium reabsorption	0.02	0.04
Apoptosis	0.02	0.04
Long-term potentiation	0.03	0.05
Type II diabetes mellitus	0.03	0.06
Huntington’s disease	0.03	0.07
VEGF signaling pathway	0.04	0.07
Arrhythmogenic right ventricular cardiomyopathy (ARVC)	0.04	0.07
**Male visceral adipose**		
Pathways in cancer	8.95E-07	7.07E-05
Jak-STAT signaling pathway	2.53E-05	9.98E-04
Pancreatic cancer	2.49E-04	0.006
Chronic myeloid leukemia	0.003	0.06
Small cell lung cancer	0.004	0.06
Bladder cancer	0.004	0.06
Pathogenic Escherichia coli infection	0.01	0.1
Acute myeloid leukemia	0.01	0.1
Huntington’s disease	0.01	0.1
Cell cycle	0.02	0.1
Focal adhesion	0.02	0.1
Tight junction	0.02	0.1
Adherens junction	0.02	0.1
TGF-beta signaling pathway	0.03	0.2
Prostate cancer	0.03	0.2
**Female subcutaneous adipose**		
Proteasome	1.76E-08	1.58E-06
Pathways in cancer	1.48E-07	6.67E-06
Adherens junction	1.43E-05	4.28E-04
Pathogenic Escherichia coli infection	1.68E-05	3.77E-04
TGF-beta signaling pathway	3.52E-05	6.33E-04
Focal adhesion	1.49E-04	0.002
Acute myeloid leukemia	1.85E-04	0.002
Prostate cancer	3.00E-04	0.003
Cell cycle	0.002	0.02
Pancreatic cancer	0.004	0.03
Chronic myeloid leukemia	0.005	0.04
Small cell lung cancer	0.008	0.05
Colorectal cancer	0.008	0.05
Thyroid cancer	0.008	0.06
Gap junction	0.01	0.06
Fc gamma R-mediated phagocytosis	0.01	0.07
Aldosterone-regulated sodium reabsorption	0.02	0.1
Wnt signaling pathway	0.02	0.1
Bladder cancer	0.02	0.1
Fc epsilon RI signaling pathway	0.03	0.1
ErbB signaling pathway	0.04	0.2
mTOR signaling pathway	0.04	0.2
Non-small cell lung cancer	0.04	0.2
**Male skeletal muscle**		
Pathways in cancer	1.49E-14	1.41E-12
Focal adhesion	1.88E-10	8.92E-09
Adherens junction	5.82E-10	1.84E-08
B cell receptor signaling pathway	5.36E-09	1.27E-07
Glioma	9.37E-09	1.78E-07
Fc epsilon RI signaling pathway	9.51E-08	1.51E-06
Pathogenic Escherichia coli infection	5.61E-07	7.61E-06
Chronic myeloid leukemia	6.52E-07	7.74E-06
Fc gamma R-mediated phagocytosis	7.44E-07	7.85E-06
ErbB signaling pathway	2.64E-06	2.51E-05
Prostate cancer	3.26E-06	2.81E-05
Natural killer cell mediated cytotoxicity	3.38E-06	2.67E-05
Melanoma	3.78E-06	2.76E-05
Non-small cell lung cancer	4.09E-06	2.78E-05
Pancreatic cancer	4.26E-06	2.70E-05
Colorectal cancer	1.55E-05	9.18E-05
T cell receptor signaling pathway	1.88E-05	1.05E-04
VEGF signaling pathway	4.88E-05	2.58E-04
Neurotrophin signaling pathway	6.25E-05	3.12E-04
Insulin signaling pathway	1.28E-04	6.09E-04
Thyroid cancer	1.39E-04	6.29E-04
Renal cell carcinoma	2.23E-04	9.64E-04
Endometrial cancer	2.92E-04	0.001
Cell cycle	3.52E-04	0.001
Acute myeloid leukemia	5.33E-04	0.002
Tight junction	5.89E-04	0.002
Bladder cancer	8.29E-04	0.003
Gap junction	9.75E-04	0.003
Type II diabetes mellitus	0.001	0.004
Regulation of actin cytoskeleton	0.001	0.004
Viral myocarditis	0.001	0.005
Jak-STAT signaling pathway	0.002	0.005
TGF-beta signaling pathway	0.004	0.01
p53 signaling pathway	0.007	0.02
MAPK signaling pathway	0.008	0.02
Notch signaling pathway	0.009	0.02
Dorso-ventral axis formation	0.009	0.02
mTOR signaling pathway	0.01	0.03
Small cell lung cancer	0.02	0.04
Leukocyte transendothelial migration	0.02	0.04
Epithelial cell signaling in Helicobacter pylori infection	0.03	0.07
Long-term depression	0.03	0.07
Aldosterone-regulated sodium reabsorption	0.04	0.08

Pathways enriched in all four conditions are shown in italics.

* Bejamini-Hochberg correction.

**Table 5:** TGF-beta signaling genes in T2D interactome-T2D transcriptome intersection.

Female visceral adipose	Male skeletal muscle
CREBBP	CDKN2B
EP300	EP300
MAPK1	MAPK1
MAPK3	MAPK3
MYC	PPP2R1B
PPP2CA	SMAD5
PPP2R1A	SP1
PPP2R1B	
ROCK1	
RPS6KB2	

**Table 6:** Enriched pathways in T2D inteactome-T2D transcriptome intersection without TGF-beta signaling genes.

Term	Unadjusted p value	Corrected p value*
**Female visceral adipose**		
Pathways in cancer	2.19E-17	2.26E-15
Prostate cancer	4.98E-13	2.57E-11
Chronic myeloid leukemia	4.58E-12	1.57E-10
Glioma	8.28E-10	2.13E-08
Small cell lung cancer	3.47E-09	7.16E-08
Proteasome	4.30E-09	7.37E-08
Pancreatic cancer	4.73E-09	6.96E-08
Non-small cell lung cancer	2.07E-08	2.66E-07
Acute myeloid leukemia	4.56E-08	5.22E-07
Melanoma	4.00E-07	4.12E-06
Neurotrophin signaling pathway	3.41E-06	3.19E-05
B cell receptor signaling pathway	5.69E-06	4.89E-05
Adherens junction	7.25E-06	5.75E-05
Jak-STAT signaling pathway	8.26E-06	6.07E-05
Endometrial cancer	1.76E-05	1.21E-04
T cell receptor signaling pathway	2.67E-05	1.72E-04
Thyroid cancer	3.65E-05	2.21E-04
Colorectal cancer	1.00E-04	5.75E-04
Cell cycle	1.04E-04	5.63E-04
Focal adhesion	1.51E-04	7.77E-04
Insulin signaling pathway	2.07E-04	0.001
Pathogenic Escherichia coli infection	2.62E-04	0.001
Bladder cancer	3.17E-04	0.001
Adipocytokine signaling pathway	7.14E-04	0.003
p53 signaling pathway	7.82E-04	0.003
Chemokine signaling pathway	9.59E-04	0.004
Fc epsilon RI signaling pathway	0.002	0.007
ErbB signaling pathway	0.003	0.01
Ubiquitin mediated proteolysis	0.003	0.01
Notch signaling pathway	0.004	0.01
Tight junction	0.01	0.03
Progesterone-mediated oocyte maturation	0.01	0.04
Apoptosis	0.01	0.04
Fc gamma R-mediated phagocytosis	0.02	0.06
Renal cell carcinoma	0.02	0.06
GnRH signaling pathway	0.02	0.06
Toll-like receptor signaling pathway	0.03	0.07
Arrhythmogenic right ventricular cardiomyopathy (ARVC)	0.03	0.07
Natural killer cell mediated cytotoxicity	0.03	0.08
Antigen processing and presentation	0.04	0.1
**Male skeletal muscle**		
Pathways in cancer	4.82E-12	4.43E-10
Focal adhesion	2.48E-09	1.14E-07
Pathogenic Escherichia coli infection	2.80E-07	8.58E-06
B cell receptor signaling pathway	3.04E-07	6.99E-06
Adherens junction	3.92E-07	7.21E-06
Glioma	6.79E-07	1.04E-05
Fc epsilon RI signaling pathway	4.28E-06	5.63E-05
Fc gamma R-mediated phagocytosis	2.20E-05	2.52E-04
Chronic myeloid leukemia	2.72E-05	2.78E-04
Natural killer cell mediated cytotoxicity	5.68E-05	5.23E-04
ErbB signaling pathway	8.02E-05	6.71E-04
Melanoma	1.48E-04	0.001
Pancreatic cancer	1.61E-04	0.001
Non-small cell lung cancer	2.33E-04	0.001
T cell receptor signaling pathway	3.65E-04	0.002
Colorectal cancer	4.23E-04	0.002
Prostate cancer	6.02E-04	0.003
Neurotrophin signaling pathway	9.21E-04	0.005
Viral myocarditis	0.001	0.005
VEGF signaling pathway	0.001	0.006
Tight junction	0.001	0.007
Insulin signaling pathway	0.002	0.007
Jak-STAT signaling pathway	0.004	0.01
Cell cycle	0.004	0.02
p53 signaling pathway	0.005	0.02
Regulation of actin cytoskeleton	0.008	0.03
Endometrial cancer	0.01	0.03
Thyroid cancer	0.01	0.04
Leukocyte transendothelial migration	0.01	0.04
Acute myeloid leukemia	0.01	0.04
Gap junction	0.01	0.04
Epithelial cell signaling in Helicobacter pylori infection	0.02	0.07
Renal cell carcinoma	0.03	0.07
MAPK signaling pathway	0.03	0.08
Bladder cancer	0.03	0.08
Arrhythmogenic right ventricular cardiomyopathy (ARVC)	0.04	0.09
Notch signaling pathway	0.04	0.1
Type II diabetes mellitus	0.04	0.1

* Bejamini-Hochberg correction.

**Table 7:** TGF-beta signaling genes in T2D genome, interactome and transcriptome, and antidiabetic drug toxicogenome.

Genome	Interactome	Transcriptome	Toxicogenome
CDKN2B	PPP2R1B	CDKN2B	BMP4
	PPP2R1A	CREBBP	DCN
	SMAD9	EP300	ID2
	ROCK1	MAPK1	IFNG
	SMAD5	MAPK3	MAPK1
	CREBBP	MYC	MAPK3
	SMAD4	PPP2CA	MYC
	SMAD3	PPP2R1A	RPS6KB1
	RPS6KB2	PPP2R1B	SP1
	RPS6KB1	ROCK1	TGFB1
	SMAD1	RPS6KB1	THBS1
	MAPK1	RPS6KB2	THBS2
	EP300	SMAD3	THBS3
	CDKN2B	SMAD5	TNF
	SP1	SP1	
	PPP2CA		
	MAPK3		
	SMURF1		
	MYC		

## Discussion

Candidate T2D genes identified in GWASs combined does not clearly confirm known aspects of disease pathophysiology. Our systems level analyses bridge this genome to phenome correlation gap. Bioinformatic analyses of disease associated genes using interactome and toxicogenome data first led us to connect T2D candidate genes identified in GWASs with disease pathophysiology including aberrant pancreatic β cell development and function, and insulin sensitivity. We then experimentally validated this connectivity using transcriptomic analysis of multiple insulin responsive tissues from male and female diabetic patients. Our simple, intuitive and straightforward approach has been remarkably successful in uncovering genome to phenome correlation in diabetes. In general, we find that candidate T2D genes identified in GWASs can explain disease pathophysiology when the associated genes are considered together with their protein and functional level interactors. In other words, the physical and genetic interaction network of T2D associated genes overall relates well with the disease pathophysiology. Our toxicogenomic analysis supports this. This evidence was finally validated in our transcriptomic analysis. It is a long standing debate whether impaired insulin action or insulin secretion deficiency is the primary defect in T2D [12], [16]. Epidemiological evidence has previously suggested that genetically programmed pancreatic β cell dysfunction interacts with environmentally triggered insulin resistance to cause T2D [12]–[14]. Our results may seem consistent with this notion.

We find tissue and gender differences in genome-wide expression profile in T2D. In males, differentially expressed genes in visceral adipose showed enrichment of valine, leucine and isoleucine degradation, and propanoate metabolism, whereas those in skeletal muscle showed enrichment of extracellular matrix-receptor interaction. In females, diverse pathways including that related to neurotrophin signaling, cancer, immune response, intercellular communication, and pathogenic *Escherichia coli* infection are enriched in differentially expressed genes in visceral adipose, whereas no pathway show enrichment in subcutaneous adipose. As such, convergence of pathways is largely absent if gene expression profiling is analyzed in isolation. However, when genes that are both differentially expressed in T2D as well as are known to interact with GWAS signals are analyzed, we find enrichment of numerous pathways in all the tissues and both genders, with several enriched pathways in common to all the conditions. Also, statistical significance of enrichment in general is greatly increased in the latter set of genes than the former. This clearly demonstrates the advantage of convergent analysis in examining genome to phenome correlation in complex disease like diabetes. Whereas interactome guided analysis of differentially expressed genes uncovered several pathophysiologically relevant pathways in female visceral adipose and male skeletal muscle, only one of them, TGF-beta signaling, was revealed, based on a literature search, in female subcutaneous adipose and male visceral adipose. This literature based analysis was supported when representatives of TGF-beta pathway were removed from the transcriptome-interactome intersection genes related to female visceral adipose and male skeletal muscle, and the resulting set was subjected to pathway enrichment analysis. Compared to complete set of intersection genes, the TGF-beta deleted list showed less pronounced enrichment of pathways including insulin signaling. Although this literature based analysis may not be very robust due to inherent limitations, and hence other pathways may also possibly be important, it at least identifies TGF-beta signaling as one that can connect genome to phenome in T2D. Of all the candidate genes identified in T2D GWASs at acceptable significance level, *CDKN2B* is the only one that represents this signaling pathway. It remains a possibility that several other genes in TGF-beta signaling are associated with the disease but we are not clear about them because they are either below the significance level used in GWAS reporting, are not yet discovered in disease association studies due to technical limitations or are not characterized and codified well enough in terms of function. A notable example here is that of *SMAD3*, a TGF-beta signaling gene. A SNP in *SMAD3* though did not show evidence of association in the original GWAS in T2D (nominal *p = *0.0006) the disease candidacy of the gene was nonetheless uncovered in a subsequent pathway enrichment analysis [62]. This supports our genome to phenome correlation analysis. The interactome network and transcriptomic analysis provided here offer novel means to mine GWAS data that is not available in public domain and identify novel candidate genes in T2D. We anticipate that new evidence for association of genes in TGF-beta pathway will emerge from data mining.

Already, some of the most recent findings do seem consistent with our systems level analysis. For example, a newly developed joint meta-analysis approach has recently identified additional loci associated with fasting insulin and other insulin resistance related traits [20]. Interestingly, genes localized nearby some of the associated SNPs are known to play a functional role in insulin signaling. Remarkably, one of the strongest positional candidates, *PPP1R3B*, is known to interact at protein level with a single gene, *PPP1CA*, which in turn is involved physically in SMAD signaling protein-protein interactions and functionally in TGF-beta signaling pathway [63]. Furthermore, we find that T2D interactome does not only include *PPP1CA* but also several of its interactors. Twenty of the total 111 *PPP1CA* interactors in the BioGRID are present in our T2D interactome of 561 genes. Given the total BioGRID space of 14,306 genes, the T2D interactome is highly enriched for *PPP1CA* interactors (*p* = 0.000000008). This demonstrates the power of our systems model. Another support for our model comes from a recently reported functional study in which the alpha-2-HS-glycoprotein (ASHG) has been identified as an adaptor protein that links saturated fatty acids to toll-like receptor 4 thus stimulating inflammatory pathways leading to insulin resistance [64]. Interestingly, ASHG is a known antagonist of TGF-beta cytokines including TGF-beta1 [65], [66]. Of the nine protein interactors of ASHG in the BioGRID, one, SMAD3, is present in our T2D interactome. Although small numbers preclude any statistical analysis, it is tempting to find this gene overlap notable.

The above discussion remarkably converges on the TGF-beta signaling effector SMAD3. TGF-beta signaling is involved in the regulation of insulin gene transcription, pancreatic islets β cell function, and glucose tolerance and energy homeostasis [36], [59]–[61]. SMAD3 is known to localize at insulin gene promoter and repress insulin gene transcription [61]. *SMAD3* knock-out mice are associated with improved glucose tolerance and insulin sensitivity [36]. Exhibiting altered expression of genes related to adipogenesis, lipid accumulation, and fatty acid β oxidation, these mice show resistance to obesity and insulin resistance induced by high fat diet [36], [59]. Further, levels of TGF-beta1 have been found to positively correlate with adiposity in human subjects [59]. Also, systemic blockade of TGF-beta signaling has been found to protect mice from obesity, diabetes and hepatic steatosis [59]. Indeed, pharmacological manipulation of TGF-beta signaling is considered to offer a potential therapeutic strategy in obesity and diabetes [59], [60].

Most recently, one of the eight new susceptibility loci reported in a large scale association analysis in T2D at genome-wide significance, the top signal maps to ZMIZ1 [67]. Notably, ZMIZ1 is known to interact with SMAD3 at protein level [68]. In the association study [67], the authors used an expanded set of susceptibility loci to define pathways and networks underlying T2D pathogenesis. Remarkably, the most connected gene that the authors found in their protein-level interaction analysis is that encoding CREBBP, a co-activator known to regulate SMAD3-dependent transcription [69]. Furthermore, the top four previously unreported T2D genes identified in a recently conducted T2D GWAS in an Indian population include the TGF-beta signaling gene TGFBR3 [70]. Known to affect phosphorylation and nuclear localization of SMAD3, TGFBR3 is involved in TGF-beta/SMAD3 dependent signaling [71]. The above results demonstrate the robustness of our systems model identifying TGF-beta/SMAD3 signaling as central to genome to phenome correlation in T2D. Above all, SNPs associated with gene expression in liver, visceral adipose and subcutaneous adipose and with T2D in GWASs have previously been found to be enriched in various R2D candidate pathways including TGF-beta signaling [72]. Overall, our global analyses seem consistent with the accumulating evidence implicating TGF-beta signaling in T2D related pathophysiology.

The newly arrived technology of whole genome/exome sequencing is expected to accelerate identification of rare variants with large effect sizes, thus helping us achieve in near future an even greater understanding of the genetic basis of complex phenotypes [2], [6]. The recently accomplished deep sequencing of human exomes has indeed suggested that rare variations contribute substantially to human phenotypic variation and disease susceptibility [73]. Availability of post-GWASs era data for T2D will be crucial in examining genome to phenome correlation in greater details. Emerging methods in pathway-wide analysis and integrative network based analysis of genetic association data in complex disorders will further help accelerate identification of variations that are causally linked to phenotypes [20], [22]. It will be interesting to see if newer results support our genome to phenome correlation analysis in general and candidacy of TGF-beta signaling in particular.

In conclusion, genetic association evidence in T2D correlates well with disease pathophysiology including insulin secretion deficiency and insulin resistance. The systems biology framework that has emerged from the present analysis may prove valuable in further refining our understanding of genetic determinants and molecular pathways in the pathogenesis of T2D.

## Materials and Methods

### Ethics Statement

All study participants provided written, informed consent under protocols specifically approved by the ethics committee of Sawai Man Singh Medical College, Jaipur.

### Associated Genes

The catalog of published GWASs made freely available by National Human Genome Research Institute was used to retrieve disease associated genes {Hindorff LA, MacArthur J (European Bioinformatics Institute), Wise A, Junkins HA, Hall PN, Klemm AK, and Manolio TA. A Catalog of Published Genome-Wide Association Studies. Available at: www.genome.gov/gwastudies. July 5, 2012}. This catalog records SNP associations from genome-wide significance level up to a *p* value threshold of 1×10^−5^ [4], [25]. It presently includes 1,300 publications and 6,581 SNPs, and contains gene names reported by the authors in the original paper. GWASs that attempted to assay at least 100,000 SNPs in the initial stage are only included in the catalog. In our analysis, we retrieved the “reported genes” in T2D GWASs using *p* value thresholds of <10^−8^, 10^−7^, 10^−6^ and 10^−5^. As our main objective was to examine correlation between genes already implicated in T2D with disease pathophysiology, we based our analysis on reported candidate genes instead of genes in linkage disequilibrium around each associated SNP.

### Pathway Enrichment

The freely available Database for Annotation, Visualization, and Integrated Discovery (DAVID) was used for examining enrichment of KEGG pathways in gene sets. DAVID, that integrates data from multiple functional databases, is frequently used for revealing biological themes underlying large gene sets (http://david.abcc.ncifcrf.gov/) [74]–[77]. Standard method of analysis was followed, with *Homo sapiens* as background and EASE score enrichment *p* values globally corrected using Benjamini-Hochberg technique [78]. A modified Fisher Exact *p* value, EASE score relate to gene-enrichment in annotation terms. KEGG, a public domain database commonly used for gene enrichment analysis and pathway visualization [22], [23], [72], [79], houses a total of 199 unique human pathways representing 5197 unique genes/proteins (http://www.genome.jp/kegg/pathway.html) [72].

### Interactome Network

The public database BioGRID extracts and annotates in-depth physical and genetic interactions reported in the primary peer-reviewed literature and houses the data that is explicitly corroborated by experimental evidence in an organized form to enable various tasks such as computational analysis of biological networks and prediction of gene/protein function (http://thebiogrid.org/) [80]–[83]. We used physical and genetic interaction data sets for *Homo sapiens* in BioGRID v.3.1.89. The total number of unique nodes and edges in these data sets were 14,306 and 67,659, respectively. To visualize BioGRID networks off-line and retrieve genes therein, we used the public domain tool Osprey (v.1.2.0) (http://biodata.mshri.on.ca/osprey/servlet/Index) [84]. Only direct interactions were used in the present analysis.

### Drug-gene Interactions

Genes that interact with antidiabetic drugs pioglitazone, troglitazone, rosiglitazone, metformin, tolbutamide, glyburide, glipizide, gliclazide, nateglinide, repaglinide, sitagliptin, saxagliptin, bromocriptine, acarbose, vildagliptin, liraglutide and exenatide were identified using the publicly available CTD, Mount Desert Island Biological Laboratory, Salisbury Cove, Maine (http://ctdbase.org/) in June, 2012. CTD is a repository of manually curated chemical-gene, chemical-disease and gene-disease relationships from the literature [85]–[86]. The database, documenting over 200,000 gene-environment relationships from over 26,000 publications, has been used previously towards understanding T2D etiology [24]. We used chemical-gene interaction query for each drug in CTD, and retrieved genes using the default settings “increases, decreases, affects” and “any” interaction. These interactions are of various types such as expression, abundance and activity.

### Surgical Tissue Samples

Biopsies were obtained from abdominal subcutaneous and visceral fat tissue and skeletal muscles of patients undergoing abdominal surgery under general anesthesia. The muscle samples were obtained from the vastus lateralis by Bergstrom needle biopsy. Primary indications of surgery were non infective and non malignant conditions, namely, cholelethiasis, hernia and trauma. The average age of the patients undergoing surgery was 58 years (range 37 to 85 years). Antidiabetic therapy mostly included sulphonylurea or insulin treatment. None of the patients were on pioglitazone ever. Once surgery was planned, all the diabetic patients were put on insulin therapy. No patient took metformin after surgery was planned. The levels of glycated hemoglobin (%HbA_1c_) determined in nondiabetic and diabetic patients were 5.75±0.33 and 9.44±0.82, respectively. The difference between the two groups was significant (*p* = 0.003). The BMI of nondiabetic and diabetic patients were 24.48±1.2 and 25.00±1.81, respectively. The difference between the two groups was insignificant (*p* = 0.81). The biopsy samples were obtained from tissue exposed and wasted during surgery. The sample was immediately rinsed in saline and stored in RNA later (Ambion, Austin, TX) solution, initially at 4°C and later stored at −80°C till further use. All study participants provided written, informed consent under protocols originally approved by the ethics committee of Sawai Man Singh Medical College, Jaipur.

### RNA Isolation

The total RNA from biopsy samples was isolated using the mirVana™ miRNA Isolation Kit (Ambion). The quantity and quality of the isolated RNA were determined Nanodrop-1000 (Thermo Fischer Scientific) and Agilent 2100 Bioanalyzer (Agilent Technologies), respectively. RNA with RIN (RNA integrity number) value in the range of 5 to 8 was used for further analysis. These RNA samples had both 260/280 nm absorbance and 28S/18S rRNAs peak ratio of two. Although RIN of 7 or more would have been ideal, RNA isolated from surgical samples may not always be of very high integrity. As biopsy specimens were limited, the RIN cutoff was lowered to 5. Notably, experiments have shown that RNA with lower RIN, i.e., with some degradation, can actually be used to perform gene expression analysis of surgical samples [87], [88].

### Microarrays

Three biological replicates with four technical replicates each were used to generate expression profiles. The biological replicates were matched with respect to gender, average age, average height, average weight, average edible oil consumption, and vegetarian/non-vegetarian diet. Also, all the patients were free from alcohol use and smoking. Starting with 500 ng of total RNA, Illumina TotalPrepTM RNA Amplification Kit (Ambion) was used for preparing first and second strand cDNA, purification of cDNA, *in vitro* transcription to synthesize biotin labeled cRNA, and purification of the labeled cRNA, in that sequence. The quantitation of cRNA was performed using Nanodrop-1000. Illumina HumanHT-12 v3 Expression BeadChip arrays, containing more than 48,000 probes representing more than 25,000 annotated genes, were hybridized with 750 ng of labeled cRNA samples. Hybridization and washing were performed according to the manufacturer’s protocol. The arrays were scanned and read using Illumina iScan System, and the data extraction, average normalization and downstream analysis performed using Illumina GenomeStudio V2010.1. IIlumina custom error model was applied to identify differentially expressed genes, with or without Benjamini and Hochberg correction for multiple hypotheses testing, as mentioned in the results section. The upregulated or downregulated genes were retrieved using ±13 Diff score threshold of Illumina custom algorithm. This Diff score corresponds to a *p* value of 0.05. Dendrogram of sample groups was constructed in GenomeStudio using globally normalized gene expression data. Correlation algorithm was used to construct the dendrogram.

### Quantitative Real Time PCR

Three biological and two to six technical replicates were used for quantitative real time PCR (qRT-PCR) analysis. The same source of RNA that was used previously for microarray analysis was used for qRT-PCR. Notably, validation of same samples used for microarray analyses is typically reported [89], [90]. The amplification reactions were carried out in 7500 Real Time PCR System (Applied Biosystems). RNA was reverse transcribed into cDNA using High capacity cDNA Archive kit (Applied Biosystems) following manufacturer’s recommendations. With 50 ng of RNA, PCR was carried out using Custom TaqMan® Array Standard 96 well Plates. Each assay consisted of two sequence-specific PCR primers and a TaqMan assay-FAM™ dye-labeled MGB probe. 18S rRNA was used as an endogenous control. Data was generated using software SDS 2.1 and C_T_ values were calculated. All genes were detectable under the detection thresholds (C_T_≤36) recommended by Applied Biosystems. To compare 18S rRNA and target gene, relative quantification was performed using comparative C_T_ method. Briefly, this method involved averaging duplicate samples of each target and endogenous control in both calibrator (i.e. control) and treatment samples [i.e. ΔC_T_ (absolute C_T_ value – endogenous control C_T_ value) and ΔΔ C_T_ (ΔC_T_ for each gene – ΔC_T_ for a common reference gene)]. The fold change was calculated according to the formula 2^−(ΔΔCT)^, where ΔΔ*C*T was the difference between ΔC_T_ target and the ΔC_T_ calibrator value. ABI gene expression assay IDs and the corresponding genes used in qRT-PCR validation experiments are listed in **Dataset S5**.

### Gene Overlap Significance Test

Common genes between two sets were identified using freely available Bioinformatics & Research Computing tool (http://jura.wi.mit.edu/bioc/tools/compare.php). Hypergeometric distribution probability was used for testing significance of gene matching. The *p* values for gene overlaps were obtained using Microsoft Office Excel 2007. Bonferroni correction was applied for adjusting *p* values for multiple hypotheses testing.

### Accession Numbers

The microarray data is available in Gene Expression Omnibus (http://www.ncbi.nlm.nih.gov/geo/), with the accession numbers GSE29221, GSE29226 and GSE29231.

## Supporting Information

Dataset S1 **Genes reported in T2D GWASs at different levels of significance.** This table lists all the genes that are reported in various GWASs for T2D at *p* value thresholds of 10^−8^, 10^−7^, 10^−6^ and 10^−5^. There are 46, 62, 71 and 93 genes in these lists, in that order. The gene lists were retrieved from the catalog of published GWASs made freely available by NHGRI.
(XLS)

Dataset S2 **Genes in the total interactome and T2D interactome.** This table lists all the genes that constitute the total interactome and the direct interaction network of genes reported in T2D GWASs at *p* value cutoff of 10^−5^. These genes are 14,306 and 561 in number, in that order. The interactome data for *Homo sapiens* in BioGRID was used in combination with the visualization tool Osprey to retrieve gene lists.
(XLS)

Dataset S3 **Antidiabetic drug interacting genes.** This table provides list of genes which interact with antidiabetic drugs pioglitazone, troglitazone, rosiglitazone, metformin, tolbutamide and glyburide. There are 121, 172, 518, 76, 17 and 23 genes in these lists, in that order. The gene lists were retrieved from Comparative Toxicogenomics Database (CTD).
(XLS)

Dataset S4 **Up- and down- regulated genes in T2D patients compared to nondiabetic controls.** This table lists genes that showed up- or down- regulation in genome-wide expression analysis in various tissues in male and/or female T2D patients. Lists of differentially expressed genes in male skeletal muscle, female subcutaneous adipose, female visceral adipose and male visceral adipose are given for both adjusted and unadjusted Diff score cutoff of ± 13 which corresponds to a *p* value of 0.05. At adjusted significance level, there are 46 up- and 62 down- regulated genes in male skeletal muscle, 200 up- and 994 down- regulated genes in female subcutaneous adipose, 1659 up- and 403 down- regulated genes in female visceral adipose, and 113 up- and 89 down- regulated genes in male visceral adipose. At unadjusted significance level, there are 773 up- and 3625 down- regulated genes in male skeletal muscle, 1175 up- and 2929 down- regulated genes in female subcutaneous adipose, 3713 up- and 1500 down- regulated genes in female visceral adipose, and 2043 up- and 1191 down- regulated genes in male visceral adipose.
(XLS)

Dataset S5 **ABI gene expression assay IDs used in qRT-PCR.** This table lists ABI gene expression IDs and the corresponding gene symbols used in qRT-PCR analysis. There are 48 IDs including that corresponding to the endogenous control 18S rRNA.
(XLS)
